# Correction to: PIMO: pathway-based interpretable multiomics interactions for multiomics integration

**DOI:** 10.1093/bioinformatics/btag657

**Published:** 2026-09-05

This is a correction to: Sai Phani Parsa, Sai Chandra Kosaraju, Euiseong Ko, Beomsu Baek, Tesfaye B Mersha, Mingon Kang, PIMO: pathway-based interpretable multiomics interactions for multiomics integration, *Bioinformatics*, Volume 42, Issue Supplement_1, July 2026, btag238, https://doi.org/10.1093/bioinformatics/btag238

In the originally published version of this manuscript, author name Tesfaye B Mersha was misspelled.

This error has been corrected.

