# Supplementary material for: PIMO: pathway-based interpretable multiomics interactions for multiomics integration
Source: Bioinformatics. 2026 Jul 7;42(Suppl 1):btag238. doi: 10.1093/bioinformatics/btag238 (PMC13340180; doi:10.1093/bioinformatics/btag238)
Supplement: btag238_Supplementary_Data [file btag238_supplementary_data.pdf]

# PIMO: Pathway-based Interpretable Multi-Omics interactions for multi-omics integration

Sai Phani Parsa[1], Sai Chandra Kosaraju[2], Euiseong Ko[3], Beomsu Baek[1], Tesfaye B. Marsha[4*], and Mingon Kang[1*]

[1] Department of Computer Science, University of Nevada, Las Vegas, NV, USA

[2] Computer Science Department, Cal Poly Pomona, CA, USA

[3] Department of Biomedical Informatics and Data Science, Heersink School of Medicine, University of Alabama at Birmingham, AL, USA

[4] Department of Medicine, Indiana University of School of Medicine, IN, USA

SPP: sai.parsa@unlv.edu

SCK: skosaraju@cpp.edu

EK: koe@uab.edu

BB: beomsu.baek@unlv.edu

TFB: tmersha@iu.edu

MK: mingon.kang@unlv.edu

*Corresponding authors: Tesfaye B. Marsha and Mingon Kang

# Supplementary information

**Supplementary Notes** ...... 1

**Supplementary Note 1: TCGA data collection and preprocessing** ...... 1

**Supplementary Note 2: Evaluation metrics** ...... 2

**Supplementary Note 3: Comparison with linear models using statistically selected features** ...... 2

**Supplementary Note 4: Computational complexity analysis** ...... 3

**Supplementary Table 1. Survival analysis performance across TCGA datasets for single-omics models, measured by C-index (mean ± standard deviation)** ...... 3

**Supplementary Table 2. Survival analysis performance across TCGA datasets for single-omics models, measured by AUC (mean ± standard deviation)** ...... 3

**Supplementary Table 3. Survival analysis performance on the METABRIC-BRCA dataset under external validation (model trained on TCGA-BRCA) and Monte Carlo cross-validation, measured by C-index (mean ± standard deviation)** ...... 4

**Supplementary Table 4: Survival analysis performance on the METABRIC-BRCA dataset under external validation (model trained on TCGA-BRCA) and Monte Carlo cross-validation, measured by AUC (mean ± standard deviation)** ...... 4

**Supplementary Table 5. Performance of Cox-EN with statistically selected features (TCGA-BRCA)** ...... 4

**Supplementary Table 6: Computational characteristics of multi-omics models, including number of parameters, training runtime, and GPU memory usage under a fixed training budget.** ...... 5

**Supplementary Table 7. Interaction effects of pathway-based gene-wise inter-omics modeling on survival analysis performance, measured by C-index (average ± std).** ...... 5

**References** ...... 6

## SUPPLEMENTARY NOTES

### SUPPLEMENTARY NOTE 1: TCGA DATA COLLECTION AND PREPROCESSING

We evaluated PIMO on five cancer cohorts from The Cancer Genome Atlas (TCGA): brain lower-grade glioma (LGG), breast invasive carcinoma (BRCA), liver hepatocellular carcinoma (LIHC), kidney renal clear cell carcinoma (KIRC), and lung adenocarcinoma (LUAD). All datasets were obtained from the TCGA Firehose Legacy cohorts via cBioPortal (cBioPortal for Cancer Genomics, 2012). Multi-omics data comprised mRNA gene expression, DNA methylation from the Illumina HumanMethylation450 (HM450) platform, and copy number alteration (CNA) data, where methylation values represent gene-level measurements aggregated from multiple CpG probes.

For each omics modality, we indexed features by Hugo gene symbols, transposed the data to a samples-by-genes layout, removed genes with invalid identifiers or entirely missing values, resolved duplicate gene entries by averaging, and applied mean imputation where necessary. We normalized each modality using z-score normalization, with parameters estimated from the training set in each Monte Carlo run and applied to the corresponding validation and test splits. Clinical data were reduced to patient and sample identifiers, overall survival time, and survival status, encoded as a binary variable (0 = censored/alive, 1 = event/deceased), with samples missing survival times excluded.

Pathway annotations were obtained from the Kyoto Encyclopedia of Genes and Genomes (KEGG, 2025). From an initial set of 365 human pathways, we removed disease-specific pathways to avoid bias toward disease-specific annotations and to focus on general biological processes, yielding 257 pathways. We further filtered by gene set size, retaining pathways with 15–300 genes, for a final set of 231 pathways.

Using these 231 pathways, we restricted all omics datasets to genes present in the selected pathways, with the gene expression gene set serving as the reference. Missing genes in the methylation and CNA data were set to 0 to ensure consistent feature dimensions across modalities. The three omics datasets were then merged by sample identifier and integrated with clinical information for downstream analysis.

For external validation, we used the METABRIC-BRCA dataset. Gene features were aligned to those used in the TCGA-BRCA training data, retaining only genes present in the TCGA-derived feature set to ensure consistency across datasets. Each omics modality was then normalized using z-score parameters estimated from the corresponding TCGA-BRCA training set in each Monte Carlo run, which were applied directly to the METABRIC data without refitting.

## SUPPLEMENTARY NOTE 2: EVALUATION METRICS

We evaluated model performance using the concordance index (C-index) and the time-dependent area under the curve (AUC) (Li et al., 2025). The C-index measures overall discriminative ability by assessing whether samples with shorter survival times receive higher predicted risk scores. To evaluate discrimination at specific time horizons, we computed AUC at 1-, 5-, and 10-year time points, comparing pairs of samples whose survival times fall on opposite sides of each threshold and measuring how often the model correctly assigns higher risk to the earlier-event sample.

## SUPPLEMENTARY NOTE 3: COMPARISON WITH LINEAR MODELS USING STATISTICALLY SELECTED FEATURES

We compared PIMO against a Cox elastic net (Cox EN) (Simon et al., 2011), baseline trained on statistically selected features from the TCGA BRCA dataset, to assess whether pathway-level multi-omics modeling provides advantages beyond standard feature selection. For each Monte Carlo split, we selected features by comparing long-term survivors (≥ 60 months) with non-long-term survivors (< 60 months), using the Wilcoxon rank-sum test for gene expression and DNA methylation, and Fisher's exact test for copy number alterations. The top $K \in \{50, 100, 200\}$ features ranked by FDR-adjusted p-values were used to train the model.

The Cox EN baseline achieved moderate performance, with gene expression consistently outperforming the other modalities (Supplementary Table 5). Increasing the number of selected features improved validation performance but did not transfer to test or external data, suggesting overfitting. Copy number alteration features showed near random performance, with only 17 features satisfying the selection criteria, indicating a limited independent prognostic signal for this modality.

PIMO outperformed the Cox EN baseline across internal and external evaluations, demonstrating that the prognostic signal in multi-omics data is not adequately captured by feature-level selection alone. Individually significant genes, when combined linearly, fail to capture cross-omics interactions that drive survival outcomes, whereas PIMO's pathway-informed architecture explicitly models these interactions within biological pathways.

## SUPPLEMENTARY NOTE 4: COMPUTATIONAL COMPLEXITY ANALYSIS

We compared PIMO, GraphPath (Ma and Wang,2024), PCLSurv (Li et al., 2025), and DeepKEGG (Lan et al., 2024) in terms of trainable parameters, training runtime, and GPU memory usage. All models were trained on NVIDIA A30 GPUs using the same data split and a fixed budget of 200 epochs.

PIMO achieved a favorable balance between predictive performance and computational cost, completing training in 353.2 s with ~15.8 GB memory (Supplementary Table 6). By comparison, GraphPath (4.88M parameters) required the highest GPU memory (~21.5 GB) and 1388.9 s despite having fewer parameters than PIMO (6.72M), while PCLSurv (7.73M) required the longest training time (4665.5 s). DeepKEGG was the most efficient (0.19M parameters, 216.9 s, ~0.8 GB) but achieved lower predictive performance.

## SUPPLEMENTARY TABLE 1. SURVIVAL ANALYSIS PERFORMANCE ACROSS TCGA DATASETS FOR SINGLE-OMICS MODELS, MEASURED BY C-INDEX (MEAN ± STANDARD DEVIATION)

| Dataset | RSF | | | Cox-EN | | | DeepSurv | | |
|---|---|---|---|---|---|---|---|---|---|
| | G | DM | CNA | G | DM | CNA | G | DM | CNA |
| LGG | 0.83±0.05 | 0.81±0.04 | 0.77±0.10 | 0.81±0.06 | 0.81±0.05 | 0.75±0.13 | 0.65±0.04 | 0.59±0.10 | 0.63±0.09 |
| KIRC | 0.71±0.09 | 0.73±0.08 | 0.60±0.10 | 0.70±0.08 | 0.73±0.07 | 0.60±0.08 | 0.64±0.07 | 0.66±0.08 | 0.55±0.07 |
| BRCA | 0.67±0.07 | 0.63±0.08 | 0.56±0.12 | 0.67±0.10 | 0.62±0.15 | 0.48±0.12 | 0.53±0.08 | 0.57±0.05 | 0.50±0.08 |
| LIHC | 0.65±0.07 | 0.64±0.07 | 0.54±0.07 | 0.60±0.08 | 0.62±0.10 | 0.52±0.10 | 0.50±0.11 | 0.55±0.09 | 0.50±0.09 |
| LUAD | 0.63±0.06 | 0.60±0.06 | 0.55±0.10 | 0.67±0.08 | 0.60±0.09 | 0.49±0.10 | 0.53±0.06 | 0.51±0.06 | 0.48±0.06 |

Note: G denotes gene expression, DM denotes DNA methylation, and CNA denotes copy number alteration.

## SUPPLEMENTARY TABLE 2. SURVIVAL ANALYSIS PERFORMANCE ACROSS TCGA DATASETS FOR SINGLE-OMICS MODELS, MEASURED BY AUC (MEAN ± STANDARD DEVIATION)

| Dataset | RSF | | | Cox-EN | | | DeepSurv | | |
|---|---|---|---|---|---|---|---|---|---|
| | G | DM | CNA | G | DM | CNA | G | DM | CNA |
| LGG | 0.86±0.06 | 0.84±0.07 | 0.78±0.10 | 0.83±0.08 | 0.86±0.04 | 0.75±0.14 | 0.66±0.09 | 0.61±0.12 | 0.66±0.10 |
| KIRC | 0.76±0.12 | 0.78±0.11 | 0.63±0.14 | 0.77±0.12 | 0.78±0.10 | 0.62±0.12 | 0.69±0.10 | 0.68±0.11 | 0.57±0.08 |
| BRCA | 0.69±0.11 | 0.66±0.11 | 0.59±0.12 | 0.68±0.10 | 0.65±0.17 | 0.47±0.17 | 0.54±0.11 | 0.61±0.05 | 0.50±0.11 |
| LIHC | 0.68±0.14 | 0.64±0.13 | 0.58±0.11 | 0.64±0.10 | 0.63±0.15 | 0.55±0.16 | 0.54±0.13 | 0.56±0.11 | 0.50±0.13 |
| LUAD | 0.63±0.09 | 0.60±0.08 | 0.60±0.13 | 0.71±0.11 | 0.61±0.12 | 0.51±0.14 | 0.55±0.10 | 0.53±0.12 | 0.52±0.09 |

Note: G denotes gene expression, DM denotes DNA methylation, and CNA denotes copy number alteration.

### Supplementary Table 3. Survival analysis performance on the METABRIC-BRCA dataset under external validation (model trained on TCGA-BRCA) and Monte Carlo cross-validation, measured by C-index (mean ± standard deviation)

| Evaluation Setting | RSF | | | Cox-EN | | | DeepSurv | | |
|---|---|---|---|---|---|---|---|---|---|
| | G | DM | CNA | G | DM | CNA | G | DM | CNA |
| External Validation | 0.57 ±0.01 | 0.50 ±0.02 | 0.56 ±0.01 | 0.54 ±0.02 | 0.53 ±0.02 | 0.52 ±0.02 | 0.50 ±0.01 | 0.51 ±0.01 | 0.51 ±0.02 |
| Monte Carlo cross-validation | 0.61 ±0.03 | 0.59 ±0.02 | 0.58 ±0.04 | 0.61 ±0.04 | 0.60 ±0.03 | 0.53 ±0.04 | 0.53 ±0.04 | 0.52 ±0.02 | 0.51 ±0.01 |

Note: G denotes gene expression, DM denotes DNA methylation, and CNA denotes copy number alteration.

### Supplementary Table 4: Survival analysis performance on the METABRIC-BRCA dataset under external validation (model trained on TCGA-BRCA) and Monte Carlo cross-validation, measured by AUC (mean ± standard deviation)

| Evaluation Setting | RSF | | | Cox-EN | | | DeepSurv | | |
|---|---|---|---|---|---|---|---|---|---|
| | G | DM | CNA | G | DM | CNA | G | DM | CNA |
| External Validation | 0.60 ±0.01 | 0.50 ±0.03 | 0.59 ±0.01 | 0.81 ±0.06 | 0.57 ±0.03 | 0.53 ±0.02 | 0.51 ±0.02 | 0.51 ±0.02 | 0.51 ±0.03 |
| Monte Carlo cross-validation | 0.66 ±0.04 | 0.62 ±0.04 | 0.60 ±0.05 | 0.65 ±0.06 | 0.53 ±0.04 | 0.54 ±0.06 | 0.54 ±0.04 | 0.53 ±0.01 | 0.51 ±0.01 |

Note: G denotes gene expression, DM denotes DNA methylation, and CNA denotes copy number alteration.

### Supplementary Table 5. Performance of Cox-EN with statistically selected features (TCGA-BRCA)

| K-value | Modality | Test C-Index | Test AUC | Eval C-Index | Eval AUC |
|---|---|---|---|---|---|
| 50 | Gene expression | 0.68 ± 0.09 | 0.69 ± 0.09 | 0.57 ± 0.01 | 0.61 ± 0.02 |
| | DNA methylation | 0.64 ± 0.12 | 0.70 ± 0.11 | 0.51 ± 0.01 | 0.51 ± 0.02 |
| | Copy number alteration | 0.51 ± 0.03 | 0.51 ± 0.04 | 0.51 ± 0.01 | 0.51 ± 0.02 |
| 100 | Gene expression | 0.65 ± 0.10 | 0.66 ± 0.12 | 0.56 ± 0.03 | 0.60 ± 0.04 |
| | DNA methylation | 0.63 ± 0.15 | 0.67 ± 0.18 | 0.52 ± 0.02 | 0.53 ± 0.03 |
| | Copy number alteration | 0.51 ± 0.03 | 0.51 ± 0.04 | 0.51 ± 0.01 | 0.51 ± 0.02 |

| | | | | | |
|---|---|---|---|---|---|
| 200 | Gene expression | 0.66 ± 0.07 | 0.68 ± 0.09 | 0.57 ± 0.02 | 0.60 ± 0.04 |
| | DNA methylation | 0.64 ± 0.16 | 0.67 ± 0.17 | 0.52 ± 0.02 | 0.53 ± 0.03 |
| | Copy number alteration | 0.51 ± 0.03 | 0.51 ± 0.04 | 0.51 ± 0.01 | 0.51 ± 0.02 |

**SUPPLEMENTARY TABLE 6: COMPUTATIONAL CHARACTERISTICS OF MULTI-OMICS MODELS, INCLUDING NUMBER OF PARAMETERS, TRAINING RUNTIME, AND GPU MEMORY USAGE UNDER A FIXED TRAINING BUDGET.**

| Model | Number of model parameters | Run time (seconds) | GPU memory usage (GiB) |
|---|---|---|---|
| DeepKEGG | 193,795 | 216.90 | 0.80 |
| GraphPath | 4,880,104 | 1388.93 | 21.48 |
| **PIMO (Ours)** | **6,717,408** | **353.19** | **15.78** |
| PCLSurv | 7,733,990 | 4665.51 | 2.80 |

**SUPPLEMENTARY TABLE 7. INTERACTION EFFECTS OF PATHWAY-BASED GENE-WISE INTER-OMICS MODELING ON SURVIVAL ANALYSIS PERFORMANCE, MEASURED BY C-INDEX (AVERAGE ± STD).**

| Dataset | Gene-wise inter-omics interactions | C-index (mean ± std) |
|---|---|---|
| LGG | | 0.83 ± 0.05 |
| | ✓ | **0.85 ± 0.03** |
| KIRC | | 0.75 ± 0.07 |
| | ✓ | 0.75 ± 0.07 |
| BRCA | | 0.65 ± 0.10 |
| | ✓ | **0.70 ± 0.08** |
| LIHC | | 0.66 ± 0.08 |
| | ✓ | **0.69 ± 0.06** |
| LUAD | | 0.64 ± 0.08 |
| | ✓ | **0.66 ± 0.06** |

Note: A check mark (✓) indicates that gene-wise inter-omics interaction modeling is enabled, else it is disabled.

# REFERENCES

cBioPortal for Cancer Genomics. cbioportal, 2012. URL https://www.cbioportal.org/. Accessed: October 2025

Kyoto Encyclopedia of Genes and Genomes. KEGG PATHWAY Database: Homo sapiens, 2025. URL https://www.kegg.jp/kegg-bin/show_organism?menu_type=pathway_maps&org=hsa. Accessed: 2025-01-20.

Zhimin Li, Chen, et al. Pclsurv: a prototypical contrastive learning-based multi-omics data integration model for cancer survival prediction. Briefings in Bioinformatics, 26(2): bbaf124, 03 2025. ISSN 1477-4054. doi: 10.1093/bib/bbaf124.

Noah Simon et al. Regularization paths for cox's proportional hazards model via coordinate descent. Journal of statistical software, 39:1–13, 2011.

Teng Ma and Jianxin Wang. Graphpath: a graph attention model for molecular stratification with interpretability based on the pathway–pathway interaction network. Bioinformatics, 40(4):btae165, 03 2024. ISSN 1367-4811. doi: 10.1093/bioinformatics/btae165.

Wei Lan et al. Deepkegg: a multi-omics data integration framework with biological insights for cancer recurrence prediction and biomarker discovery. Briefings in Bioinformatics, 25(3):bbae185, 04 2024. ISSN 1477-4054.doi: 10.1093/bib/bbae185.
